# The Dioradin Treatment

**Published:** 1912-08-10

**Authors:** 


### THE DIORADIN TREATMENT.

Some time ago some French and other Continental authors made much of a new treatment of tuberculosis known as the dioradin method. The claims put forward appeared so marvellous that in The Hospital we felt compelled to urge the need for caution in accepting them, and for a suspension of judgment until they had been independently tested. Shortly after that warning, the medical profession in this country was inundated with pamphlets containing extracts from papers by foreign physicians extolling the wonderful cures effected by dioradin; and all the wiles of the enterprising drug manufacturer were employed to push the new remedy. As usual, this commercial activity induced a good many doctors in practice—too busy themselves to investigate the matter in a scientific way—to give dioradin a trial; but no general conviction seems to have diffused itself of the efficacy of this method of treatment.

Fortunately, an independent and expert study of the dioradin method has been carried on at the Brompton Hospital for Consumption and Diseases of the Chest, the results of which are tabulated in a recent issue of the *British Medical Journal.* The general conclusions there reached justify the scepticism with which The Hospital felt bound to receive the extravagant claims originally made when the matter was first heard of; and since the whole episode enforces a moral which has more than once been pointed out in these columns, it may be as well to quote some of the detailed items in the considered judgment of the author in question. Ten cases in all were under treatment: nine of these were pulmonary cases, the other a case of tuberculous epididymitis and peritonitis. In one case tubercle bacilli, numerous in the sputum to begin with, were difficult to find at the end of a month, but were still present five months later: in the other eight pulmonary cases tubercle bacilli were still present as long as the patients remained under observation. In six cases improvement was recorded during treatment; but in only one was it maintained. In no case was there complete arrest of the disease. The cases were treated accurately in accordance with the instructions given; but none of them, in the opinion of the Brompton Hospital physician, can be held "to justify any claims for the therapeutic efficiency of dioradin."

Thus the much vaunted dioradin treatment joins the list of discarded remedies, already so long, which have all in their time been loudly proclaimed (by interested parties) as unfailing specifics against consumption. The rocket-like advent and eclipse of dioradin are a sequence of events unhappily so common that it would hardly be worth while to allude to them were it not for the certainty that similar exploitation of the profession and the public will be found to recur. Doctors cannot eradicate the innate credulity of the community or the tendency of consumptives to clutch, like drowning swimmers, at the frailest straw. But they can and should make more effort, as a profession, than they do to resist the importunities of the insinuating vendors of new and untried specifics for any and every disease under the sun. Unfortunately, to adopt energetically the latest fad and to run it to death until another displaces it is a reasonably sure way to amass money, and since a glib tongue and a dormant conscience are all the professional equipment necessary it is not to be wondered at that a certain proportion of the profession is found to fall into the temptation, which each of these crazes offers in turn. We can only record the warning that dioradin provides in this respect.

